# Replacement of soybean meal with fermented rapeseed meal in broiler diets: impacts on growth performance, gut health, and nutrient digestibility

**DOI:** 10.1016/j.psj.2025.105616

**Published:** 2025-07-27

**Authors:** Behrouz Dastar, Amin Ashayerizadeh, Fatemeh Sharifi, Vahid Jazi

**Affiliations:** a Department of Animal and Poultry Nutrition, Faculty of Animal Science, Gorgan University of Agricultural Sciences and Natural Resources, Gorgan, Iran; b Department of Animal Science, School of Agriculture, Shiraz University, Shiraz, Iran; c Central Queensland Innovation and Research Precinct (CQIRP), Institute for Future Farming Systems, Central Queensland University, Rockhampton, QLD 4701, Australia; d Department of Physiology, School of Medicine, Isfahan University of Medical Sciences, Isfahan, Iran

**Keywords:** Broiler, Gut health, Fermented rapeseed meal, Growth performance, Lactic acid bacteria

## Abstract

The inclusion of raw rapeseed meal (**RSM**) in broiler diets is often limited due to the presence of anti-nutritional factors. Microbial fermentation has been proposed as an effective strategy to improve the nutritional value of plant-based protein sources. This study aimed to evaluate the effects of replacing soybean meal (**SBM**) with either RSM or fermented RSM (**FRSM**) on growth performance, gut microbiota, biochemical indices, intestinal morphology, nutrient digestibility, and cecal short-chain fatty acid concentrations in broiler chickens. A total of 300 one-day-old male Cobb 500 broiler chicks were randomly assigned to five dietary treatments and reared for 42 days. The experimental diets included a corn–SBM-based control diet; a diet in which SBM was entirely replaced with RSM; and three diets in which FRSM replaced RSM at 33.3 % (**FRSM_33_**), 66.6 % (**FRSM_66_**), and 100 % (**FRSM_100_**), respectively. Replacing SBM with RSM significantly reduced body weight gain and increased feed conversion ratio (*P* < 0.05). In contrast, complete substitution of SBM with FRSM mitigated these adverse effects, significantly improving growth performance compared with diets containing high levels of RSM (*P* < 0.05). The inclusion of FRSM in place of SBM significantly increased lactic acid bacteria counts in the crop and reduced coliform counts in the ileum (*P* < 0.05). Furthermore, birds receiving FRSM diets exhibited significantly lower serum concentrations of total cholesterol, triglycerides, very low-density lipoprotein cholesterol, and low-density lipoprotein cholesterol (*P* < 0.05). The jejunal villus height to crypt depth ratio and cecal concentrations of butyric and lactic acids were significantly higher in birds fed diets containing FRSM compared with those fed RSM or the SBM diet (*P* < 0.05). The apparent ileal digestibility of crude protein and ether extract was also significantly improved in FRSM diets relative to the RSM diet (*P* < 0.05). Under the conditions of this study, replacing SBM with FRSM improved growth performance, nutrient digestibility, gut microbiota composition, short-chain fatty acid concentrations, and intestinal morphology relative to RSM diets. Although FRSM did not fully match the growth performance achieved with SBM, it improved nutrient utilization and gastrointestinal health. These findings suggest that FRSM may serve as a functional alternative protein source to SBM by promoting gut integrity and supporting physiological health in broiler chickens.

## Introduction

The global poultry industry is currently facing major challenges, primarily driven by the rising costs and limited availability of soybean meal (**SBM**), which has traditionally served as the primary protein source in poultry nutrition (Elbaz et al., 2023). This reliance on SBM raises concerns regarding both economic sustainability and supply chain stability. Consequently, there is increasing interest in identifying alternative protein sources that can deliver comparable or improved nutritional value while remaining economically feasible for large-scale poultry production (Peng et al., 2022; Ashayerizadeh et al., 2024a).

Rapeseed meal (**RSM**) contains a relatively high crude protein content (34 %–38 %) and offers a well-balanced amino acid profile, making it a promising substitute for SBM in poultry diets. However, its inclusion in monogastric nutrition is limited due to the presence of anti-nutritional factors such as glucosinolates, phytic acid, and phenolic compounds (Khajali and Slominski, 2012). These compounds negatively impact nutrient utilization through various pathways, including the inhibition of digestive enzyme activity, chelation of essential minerals, and disruption of nutrient transport across the intestinal epithelium (Czech et al., 2021; Taheri et al., 2023).

To mitigate these limitations, various strategies have been explored, including supplementation with exogenous enzymes (Toghyani et al., 2009) and dietary copper (Payvastegan et al., 2013), both of which have been shown to improve nutrient digestibility and growth performance in broiler chickens. More recently, microbial fermentation has emerged as a promising biotechnological approach to enhance the nutritional quality of plant protein sources (Jazi et al., 2017). In this process, specific microbial strains are employed, including bacteria such as *Lactobacillus acidophilus, Enterococcus faecium*, and *Bacillus subtilis*; fungi such as *Aspergillus niger* and *Aspergillus oryzae*; and yeasts such as *Saccharomyces cerevisiae*, applied either individually or in combination. These microorganisms enzymatically hydrolyze complex macromolecules into simpler, more bioavailable forms such as organic acids, monosaccharides, and amino acids, thereby improving nutrient availability and feed efficiency (Olukomaiya et al., 2019; Jazi et al., 2019). Fermentation of RSM has been reported to reduce anti-nutritional factors such as glucosinolates by up to 52 % and increase crude protein content by approximately 10 % (Vlassa et al., 2022). Additionally, combined fermentation with *Bacillus subtilis* and *Saccharomyces cerevisiae* has been shown to significantly lower glucosinolate and crude fiber contents (Zhu et al., 2023). These biochemical changes are expected to alleviate the inhibitory effects of anti-nutritional factors, thereby enhancing nutrient digestibility and promoting gut health. Previous feeding trials have shown that partial replacement of SBM with fermented rapeseed meal (**FRSM**) can enhance broiler growth performance and immune function when compared to diets containing RSM (Ashayerizadeh et al., 2018; Wu et al., 2022). In addition, improvements in antioxidant status and intestinal morphology have also been reported in broilers fed FRSM diets (Hu et al., 2016). However, the consequences of completely substituting SBM with FRSM on gut health, physiological functionality, and overall productivity in broilers remain insufficiently characterized and warrant further investigation. Therefore, the present study was designed to test the hypothesis that dietary inclusion of FRSM can attenuate the adverse effects associated with RSM by reducing anti-nutritional factors (e.g., glucosinolates, phytates, and tannins), thereby enhancing nutrient digestibility and potentially enabling extensive replacement of SBM. Furthermore, based on previous findings regarding the probiotic effects of fermented feed ingredients (Jazi et al., 2018a), FRSM was also hypothesized to promote gastrointestinal health. Accordingly, the objective of this study was to evaluate and compare the effects of fully replacing SBM with either RSM or FRSM on growth performance, gut microbiota composition, serum biochemical indices, intestinal morphology, nutrient digestibility, and cecal short-chain fatty acid concentrations in broiler chickens.

## Materials and methods

The experimental protocols describing the management and care of animals were reviewed and approved by the Animal Ethics Committee of Gorgan University of Agricultural Sciences and Natural Resources, Gorgan, Iran (Approval No. AEC395p/06/27).

### Processing of FRSM

*Lactobacillus acidophilus* (PTCC 1643), *Bacillus subtilis* (PTCC 1156), and *Aspergillus niger* (PTCC 5010) were obtained from the Persian Type Culture Collection of the Iranian Research Organization for Science and Technology (IROST, Tehran, Tehran Province, Iran). Rapeseed meal was sourced from Golestan Oilseed Processing Co. (Gorgan, Golestan Province, Iran). Prior to fermentation, RSM was autoclaved at 121°C for 15 min to deactivate endogenous myrosinase enzyme. Each kilogram of autoclaved RSM was mixed with 1.2 L of sterile distilled water containing a mixed inoculum of the three microorganisms (10⁵ CFU/mL for bacteria and 10⁶ spores/mL for *Aspergillus niger*). Fermentation was conducted in anaerobic stainless-steel tanks equipped with one-way gas release valves, allowing the efflux of produced gases and preventing oxygen entry. The tanks were maintained at 30°C and 60 % relative humidity for 25 days. Subsequently, the fermented samples were spread on polyethylene sheets and dried at 30-40°C for 3 days, until they reached an approximate dry matter content of 900 g/kg. The dried material was ground to pass through a 0.5 mm sieve and stored at ambient temperature (∼22°C) until incorporation into diets. To ensure consistency and scalability, all fermented product used in feeding trials was produced in bulk under identical fermentation conditions across multiple batches.

### Chemical composition of RSM and FRSM

The chemical composition of RSM and FRSM was determined according to the standard procedures of the Association of Official Analytical Chemists (AOAC, 2005). The analyses included determination of dry matter, crude protein, ether extract, ash, and crude fiber. The phytic acid content was measured after extraction with hydrochloric acid and sodium sulfate, with absorbance recorded at 660 nm using a spectrophotometer. Total tannins and phenolic compounds were quantified using the vanillin–HCl colorimetric assay, with absorbance measured at 500 nm. Glucosinolates were enzymatically hydrolyzed using myrosinase, and the released glucose was quantified via high-performance liquid chromatography. To enumerate lactic acid bacteria (**LAB**), 1 g of each sample (RSM and FRSM) was homogenized in 9 mL of buffered peptone water and serially diluted tenfold. From appropriate dilutions, 0.1 mL aliquots were plated on modified de Man, Rogosa, and Sharpe agar and incubated anaerobically at 37°C for 24 hours. Colony-forming units (**CFU**) were counted and expressed per gram of sample. For pH measurement, 20 g of each sample was mixed with 200 mL of distilled water in a 250-mL beaker, thoroughly stirred, and the pH was recorded using a calibrated portable pH meter (Model CG 804; Schott Geräte GmbH, Mainz, Germany). The changes in chemical composition of RSM before and after fermentation are summarized in Table 1.

**Table 1:** Chemical composition of rapeseed meal pre- and post-fermentation (g/kg, dry matter basis).

Component	Rapeseed meal	
	Pre-fermentation	Post-fermentation
Dry matter, g/kg	931.2	910.8
Crude protein, g/kg	375.1	402.3
Crude fiber, g/kg	116.0	61.1
Ether extract, g/kg	15.2	12.1
Ash, g/kg	55.0	67.8
Glucosinolates, µmol/g	12.21	3.93
Phytic acid, mg/g	26.35	4.52
Tannin and phenolic compounds, mg/g	17.19	7.13
pH	5.07	3.97
Lactic acid bacteria, log_10_ CFU/g	5.44	13.55

### Broiler chicks, housing, and experimental design

A total of 300 one-day-old male broiler chicks (Cobb 500) were obtained from a commercial hatchery, weighed, and randomly assigned to five dietary treatments. Each treatment consisted of six replicates with 10 birds per replicate, and the trial lasted for 42 days. Chicks were housed in floor pens (150 × 110 cm) with ad libitum access to feed and fresh water. A continuous lighting program was implemented throughout the experimental period. The ambient temperature was initially set at 35°C for the first three days, then gradually decreased to 23°C by the fourth week, and maintained until the end of the experiment, following standard poultry management guidelines. The experimental diets included: a corn-SBM-based control diet; a diet in which SBM was entirely replaced by RSM, and three diets in which FRSM substituted RSM at 33.33 %, 66.66 %, and 100 % levels (**FRSM_33_, FRSM_66_**, and **FRSM_100_**, respectively). All diets were formulated to meet or exceed the nutritional requirements of Cobb 500 broilers (Cobb-Vantress, 2012). The ingredient and nutrient compositions of the starter (days 1–10), grower (days 11–22), and finisher (days 23–42) diets are presented in Table 2. To evaluate apparent ileal digestibility, chromic oxide was added as an indigestible marker at 3 g/kg of diet from days 32 to 42.

**Table 2:** Composition and analyzed nutrient content of experimental diets for starter (1–10 d), grower (11–22 d), and finisher (23–42 d) phases.

Item	Starter					Grower					Finisher				
Ingredients	CON	RSM	FRSM_33_	FRSM_66_	FRSM_100_	CON	RSM	FRSM_33_	FRSM_66_	FRSM_100_	CON	RSM	FRSM_33_	FRSM_66_	FRSM_100_
Ingredients, g/kg															
Corn	586.3	504.8	516.7	528.5	540.3	606.3	535.1	543.2	554.0	564.6	644.2	584.5	590.3	599.5	608.4
Soybean meal	307.1	0.0	0.0	0.0	0.0	280.3	0.0	0.0	0.0	0.0	238.7	0.0	0.0	0.0	0.0
Rapeseed meal	0.0	370.4	246.7	123.3	0.0	0.0	335.6	223.5	111.8	0.0	0.0	284.8	189.7	94.8	0.0
fermented rapeseed meal	0.0	0.0	113.2	226.1	338.8	0.0	0.0	104.8	206.9	309.2	0.0	0.0	89.8	176.6	263.3
Corn gluten meal	30.0	30.0	30.0	30.0	30.0	30.0	30.0	30.0	30.0	30.0	30.0	30.0	30.0	30.0	30.0
Fish meal	20.0	20.0	20.0	20.0	20.0	20.0	20.0	20.0	20.0	20.0	20.0	20.0	20.0	20.0	20.0
Sunflower oil	19.3	42.1	40.1	38.2	36.3	29	49.3	48.0	46.2	44.5	36	53.1	52.1	50.6	49.2
Limestone	10.1	6.6	6.7	6.8	6.9	9.6	6.4	6.4	6.5	6.6	8.8	6.1	6.2	6.2	6.3
Dicalcium phosphate	13.5	12.4	12.5	12.6	12.7	12.5	11.4	11.5	11.6	11.7	10.9	10.1	10.1	10.2	10.3
Salt	3.2	3.2	3.2	3.2	3.2	3.2	3.2	3.2	3.2	3.2	3.0	3.0	3.0	3.0	3.0
Vitamin premix^1^	2.5	2.5	2.5	2.5	2.5	2.5	2.5	2.5	2.5	2.5	2.5	2.5	2.5	2.5	2.5
Mineral premix^2^	2.5	2.5	2.5	2.5	2.5	2.5	2.5	2.5	2.5	2.5	2.5	2.5	2.5	2.5	2.5
Antioxidant	0.3	0.3	0.3	0.3	0.3	0.3	0.3	0.3	0.3	0.3	0.3	0.3	0.3	0.3	0.3
DL-Met	2.4	0.8	0.9	1.0	1.1	1.9	0.4	0.5	0.6	0.7	1.6	0.4	0.5	0.6	0.7
L-Lys	2.4	4.1	4.3	4.5	4.7	1.8	3.3	3.5	3.7	3.9	1.4	2.7	2.9	3	3.2
L-Thre	0.4	0.3	0.4	0.5	0.7	0.1	0.0	0.1	0.2	0.3	0.1	0.0	0.1	0.2	0.3
Calculated composition															
Metabolizable energy, MJ/kg	12.55	12.55	12.55	12.55	12.55	12.97	12.97	12.97	12.97	12.97	13.39	13.39	13.39	13.39	13.39
Crude protein, g/kg	210.0	210.0	210.0	210.0	210.0	200.0	200.0	200.0	200.0	200.0	185.0	185.0	185.0	185.0	185.0
Lys, g/kg	13.0	13.0	13.0	13.0	13.0	11.8	11.8	11.8	11.8	11.8	10.6	10.6	10.6	10.6	10.6
Met, g/kg	6.2	5.1	5.2	5.2	5.3	5.5	4.6	4.6	4.7	4.7	5.1	4.3	4.3	4.7	4.4
Met + Cys, g/kg	9.7	9.7	9.7	9.7	9.7	8.9	8.9	8.9	8.9	8.9	8.3	8.3	8.3	8.3	8.3
Thre, g/kg	8.5	8.5	8.5	8.5	8.5	7.8	7.8	7.8	7.8	7.8	7.1	7.1	7.1	7.1	7.1
Ca, g/kg	8.9	8.9	8.9	8.9	8.9	8.4	8.4	8.4	8.4	8.4	7.6	7.6	7.6	7.6	7.6
Available P, g/kg	4.4	4.4	4.4	4.4	4.4	4.2	4.2	4.2	4.2	4.2	3.8	3.8	3.8	3.8	3.8
Na, g/kg	1.6	1.6	1.6	1.6	1.6	1.6	1.6	1.6	1.6	1.6	1.5	1.5	1.5	1.5	1.5
Analyzed composition^3^															
Dry matter, g/kg	891.6	907.8	904.8	901.8	898.8	892.1	906.5	903.9	901.2	898.5	891.6	904.0	901.8	899.5	897.2
Crude protein, g/kg	212.2	221.7	213.0	211.0	210.5	202.3	202.4	201.8	200.9	201.5	185.9	186.2	185.8	186.1	185.5
Ether extract, g/kg	49.1	69.1	67.1	65.2	63.2	58.9	76.8	75.4	73.5	71.8	66.5	81.5	80.4	79.0	77.5
Ash, g/kg	57.5	52.4	53.6	54.9	56.1	54.7	49.7	50.8	51.9	53.0	49.7	45.7	46.7	47.6	48.5

Treatment groups included: CON: Control (basal corn-SBM diet); RSM: Rapeseed meal was replaced entirely with SBM in the control diet; FRSM_33_: Fermented rapeseed meal was replaced with rapeseed meal at 33.33 % in the RSM diet; FRSM_66_: Fermented rapeseed meal was replaced with rapeseed meal at 66.66 % in the RSM diet. FRSM_100_: Fermented rapeseed meal was replaced entirely with rapeseed meal in the RSM diet.

1 Supplied per kg of diet: 1.8 mg all-trans-retinyl acetate; 0.02 mg cholecalciferol; 8.3 mg alphatocopheryl acetate; 2.2 mg menadione; 2 mg pyridoxine HCl; 8 mg cyanocobalamin; 10 mg nicotine amid; 0.3 mg folic acid; 20 mg d-biotin; 160 mg choline chloride.

2 Supplied per kg of diet: 32 mg Mn (MnSO_4__H_2_O); 16 mg Fe (FeSO_4__7H_2_O); 24 mg Zn (ZnO); 2 mg Cu (CuSO_4__5H_2_O); 800 *µ*g I (KI); 200 *µ*g Co (CoSO_4_); 60 *µ*g Se (NaSeO_3_).

3 Dry matter, crude protein, ether extract, and ash contents were determined according to the procedures described by AOAC (2005).

### Sampling and measurements

Body weight and feed intake (**FI**) were recorded on days 10, 22, and 42 of the experimental period. Feed conversion ratio (**FCR**) was calculated as the ratio of weight gain (**WG**) to FI. On day 42, two birds per pen, selected based on the average pen body weight, were euthanized for sampling. The following parameters were evaluated: digesta pH, microbial populations in the crop and ileum, small intestinal morphology, nutrient digestibility, and concentrations of short-chain fatty acids. Following euthanasia, the gastrointestinal tract was excised, and 3-cm segments were collected from the midpoint of the duodenum, jejunum, and ileum. These segments were immediately fixed in 4 % paraformaldehyde, dehydrated through a graded ethanol series, cleared in histolene, and embedded in paraffin wax. Tissue sections (5 µm) were prepared using a microtome and stained with hematoxylin and eosin for histological analysis. Villus height and crypt depth were measured using a light microscope (Model AX70; Olympus Corporation, Tokyo, Japan) equipped with a digital video camera (Model Eclipse TS100; Nikon Corporation, Tokyo, Japan).

The pH of the crop and ileal digesta was measured using a portable pH meter (Model CG 804; Schott Geräte, Hofheim, Germany) after homogenizing 1 g of digesta with 2 mL of distilled water. For microbial enumeration, 1 g of digesta was diluted in 9 mL of sterile peptone water to prepare a series of 10-fold serial dilutions. From appropriate dilutions, 0.1 mL aliquots were plated onto modified de Man, Rogosa, and Sharpe agar for LAB, violet red bile agar for coliforms, and plate count agar for total anaerobic bacteria. The plates were incubated at 37.5°C for 24 to 48 hours, after which bacterial colonies were enumerated and expressed as CFU per gram of digesta.

For nutrient digestibility assessment, ileal digesta were collected from the distal ileum (Meckel's diverticulum to 40 mm before the ileocecal junction), pooled per pen, frozen at −20°C, dried at 65°C, ground, and analyzed for dry matter, crude protein, ether extract, and organic matter using AOAC (2005) procedures. Chromium concentration, used as an indigestible marker, was measured spectrophotometrically after acid digestion. The apparent ileal digestibility of nutrients was calculated based on nutrient-to-marker ratios in diet and digesta.

To quantify short-chain fatty acid concentrations, cecal digesta were diluted with distilled water at a 1:4 (w/v) ratio and centrifuged at 4,000 rpm for 10 minutes at 4°C. Then, 1 mL of the supernatant was mixed with 2 mL of an internal standard solution comprising 25 % metaphosphoric acid containing crotonic acid and centrifuged again at 10,000 rpm for 10 minutes at 4°C. The resulting supernatant was analyzed using gas chromatography (Model PU 4410; Philips, Amsterdam, Netherlands) following Calik and Ergun (2015), and short-chain fatty acid concentrations were expressed as µmol/g of cecal digesta.

Blood samples were collected from the wing vein of two birds per pen and centrifuged at 2,000 × *g* for 5 minutes to separate the serum, which was subsequently stored at −20°C until further analysis. Serum concentrations of total cholesterol, triglycerides, high-density lipoprotein-cholesterol (**HDL-C**), total protein, and glucose were measured using commercial laboratory kits (Pars Azmoon Kits; Pars Azmoon, Tehran, Iran). Very low-density lipoprotein-cholesterol (**VLDL-C**) was estimated by dividing the triglyceride concentration by 5, and low-density lipoprotein-cholesterol (**LDL-C**) was calculated by subtracting the sum of HDL-C and VLDL-C from the total cholesterol (Arun et al., 2006).

On day 42, carcass components, including breast, thigh, abdominal fat, liver, spleen, heart, and bursa of Fabricius, were excised post-defeathering and weighed. These values were expressed as percentages of the live body weight.

### Statistical analysis

Data were analyzed using a completely randomized design with the General Linear Model procedure in SAS software. Treatment means were compared using Tukey's HSD test, with statistical significance set at *P* < 0.05.

## Results

### Growth performance

As shown in Table 3, broilers fed the SBM-based control diet consistently exhibited the highest WG and the most favorable FCR across all growth phases (*P* < 0.05). In contrast, birds receiving the diet in which SBM was completely replaced with RSM showed the lowest WG and FI, along with the poorest FCR. Partial substitution of RSM with FRSM at 33 % and 66 % inclusion levels significantly improved growth performance compared to the full RSM group (*P* < 0.05). Moreover, complete replacement of SBM with FRSM further improved WG and FCR relative to all RSM-containing diets (*P* < 0.05), suggesting that fermentation effectively mitigated the growth-inhibitory effects of RSM. However, despite these improvements, the birds in the full FRSM group still exhibited significantly lower WG and less efficient FCR than those in the SBM control group (*P* < 0.05).

**Table 3:** Effects of dietary RSM and FRSM on the performance of broiler chickens during different growth phases.

	Treatments						
Items^1^	CON	RSM	FRSM_33_	FRSM_66_	FRSM_100_	SEM^2^	*P*-value
Starter (1-10 d)							
WG, g	140.16^a^	117.83^c^	120.08^c^	127.91^b^	130.16^b^	1.05	<0.0001
FI, g	169.91^a^	165.00^b^	166.58^b^	172.00^a^	171.58^a^	0.79	<0.0001
FCR, g/g	1.21^c^	1.40^a^	1.38^a^	1.34^b^	1.310^b^	0.01	<0.0001
Grower (11-22 d)							
WG, g	805.41^a^	683.90^c^	686.07^c^	763.91^b^	767.43^b^	11.91	<0.0001
FI, g	1242.97^a^	1222.55^b^	1224.05^b^	1250.49^a^	1249.08^a^	6.97	0.02
FCR, g/g	1.54^c^	1.78^a^	1.78^a^	1.64^b^	1.62^b^	0.02	<0.0001
Finisher (23-42 d)							
WG, g	1302.69^a^	994.52^c^	1014.81^c^	1186.06^b^	1195.10^b^	11.43	<0.0001
FI, g	2926.92^a^	2885.76^b^	2902.88^b^	2941.44^a^	2935.19^a^	5.99	<0.0001
FCR, g/g	2.24^c^	2.90^a^	2.86^a^	2.48^b^	2.45^b^	0.02	<0.0001
Total grow-out period (1-42 d)							
WG, g	2248.27^a^	1796.25^c^	1820.96^c^	2077.88^b^	2092.69^b^	13.20	<0.0001
FI, g	4339.81^a^	4273.32^b^	4293.53^b^	4363.94^a^	4355.86^a^	8.84	<0.0001
FCR, g/g	1.93^c^	2.37^a^	2.35^a^	2.10^b^	2.08^b^	0.01	<0.0001

Treatment groups included: CON: Control (basal corn-SBM diet); RSM: Rapeseed meal was replaced entirely with SBM in the control diet; FRSM_33_: Fermented rapeseed meal was replaced with rapeseed meal at 33.33 % in the RSM diet; FRSM_66_: Fermented rapeseed meal was replaced with rapeseed meal at 66.66 % in the RSM diet. FRSM_100_: Fermented rapeseed meal was replaced entirely with rapeseed meal in the RSM diet.

1 WG, weight gain; FI, feed intake; FCR, feed conversion ratio.

2 SEM, standard error of the means.

### Carcass traits

The results presented in Table 4 indicate that broilers fed the control diet had a significantly higher carcass yield compared to all other groups (*P* < 0.05). Carcass yield decreased following the replacement of SBM with RSM; however, partial or complete replacement of SBM with FRSM improved carcass yield relative to the RSM group (*P* < 0.05). Similarly, breast and thigh muscle yields were significantly higher in broilers fed the control, FRSM_66_, and FRSM diets compared to those fed the RSM and FRSM_33_ diets (*P* < 0.05). Dietary inclusion of FRSM, whether partial or complete, significantly reduced abdominal fat yield compared to both RSM and SBM diets (*P* < 0.05). In contrast, replacing SBM with either RSM or FRSM significantly increased liver yield (*P* < 0.05). No differences were observed in the relative weights of the spleen, heart, or bursa of Fabricius among the treatment groups (*P* > 0.05).

**Table 4:** Effects of dietary RSM and FRSM on carcass characteristics of broiler chicks on day 42.

	Treatments						
Items^1^	CON	RSM	FRSM_33_	FRSM_66_	FRSM_100_	SEM^2^	*P*-value
Carcass, % LBW	64.40^a^	55.86^c^	56.66^c^	59.89^b^	60.61^b^	1.02	0.0002
Breast, % LBW	24.44^a^	20.95^b^	21.27^b^	24.43^a^	24.65^a^	0.56	<0.0001
Thigh, % LBW	20.89^a^	18.85^b^	19.11^b^	21.56^a^	21.57^a^	0.48	<0.0001
Abdominal fat, % LBW	2.16^a^	1.95^a^	1.70^ab^	1.32^b^	1.18^b^	0.17	0.005
Liver, % LBW	1.99^b^	2.87^a^	2.91^a^	2.88^a^	2.70^a^	0.10	0.009
Spleen, % LBW	0.13	0.13	0.14	0.13	0.14	0.01	0.49
Heart, % LBW	0.59	0.65	0.66	0.63	0.62	0.02	0.29
Bursa of Fabricius, % LBW	0.10	0.10	0.12	0.11	0.11	0.006	0.43

Treatment groups included: CON: Control (basal corn-SBM diet); RSM: Rapeseed meal was replaced entirely with SBM in the control diet; FRSM_33_: Fermented rapeseed meal was replaced with rapeseed meal at 33.33 % in the RSM diet; FRSM_66_: Fermented rapeseed meal was replaced with rapeseed meal at 66.66 % in the RSM diet. FRSM_100_: Fermented rapeseed meal was replaced entirely with rapeseed meal in the RSM diet.

1 LBW, live body weight.

2 SEM, standard error of the means.

### pH and bacterial counts

According to the data presented in Table 5, substitution of SBM with FRSM at all inclusion levels significantly reduced pH values in both the crop and the ileum compared to birds fed the RSM or control diets (*P* < 0.05). Total anaerobic bacterial counts were also significantly lower in all FRSM-fed groups relative to the RSM and control groups (*P* < 0.05). In addition, replacing SBM with FRSM significantly increased the population of LAB in the crop (*P* < 0.05). Furthermore, the lowest coliform counts in the ileum were observed in the FRSM groups (*P* < 0.05).

**Table 5:** Effects of dietary RSM and FRSM on gut microbial populations and digesta pH values in broiler chicks on day 42.

	Treatments						
Items^1^	CON	RSM	FRSM_33_	FRSM_66_	FRSM_100_	SEM^2^	*P*-value
In crop							
pH	4.59^a^	4.62^a^	4.30^b^	4.28^b^	4.25^b^	0.03	<0.0001
TAB, log_10_ CFU/g	6.49^b^	6.66^a^	6.32^c^	6.24^c^	6.21^c^	0.05	0.0002
LAB, log_10_ CFU/g	7.77^b^	7.51^c^	7.89^ab^	7.96^a^	8.02^a^	0.05	<0.0001
In ileum							
pH	6.39^a^	6.46^a^	6.28^b^	6.23^b^	6.21^b^	0.02	<0.0001
TAB, log_10_ CFU/g	6.17^b^	6.30^a^	6.08^bc^	6.03^c^	5.98^c^	0.03	<0.0001
Coliforms, log_10_ CFU/g	4.06^b^	4.21^a^	3.87^c^	3.80^c^	3.76^c^	0.03	<0.0001

Treatment groups included: CON: Control (basal corn-SBM diet); RSM: Rapeseed meal was replaced entirely with SBM in the control diet; FRSM_33_: Fermented rapeseed meal was replaced with rapeseed meal at 33.33 % in the RSM diet; FRSM_66_: Fermented rapeseed meal was replaced with rapeseed meal at 66.66 % in the RSM diet. FRSM_100_: Fermented rapeseed meal was replaced entirely with rapeseed meal in the RSM diet.

1 TAB, total anaerobic bacteria; LAB, lactic acid bacteria.

2 SEM, standard error of the means.

### Serum biochemical values

Table 6 presents the serum biochemical parameters of broiler chickens in response to the dietary treatments. Birds fed FRSM-containing diets exhibited significantly lower serum concentrations of total cholesterol, triglycerides, LDL-C, and VLDL-C compared to those fed RSM or SBM diets (*P* < 0.05). The concentrations of HDL-C, total protein, and glucose did not differ among the dietary treatment groups (*P* > 0.05).

**Table 6:** Effects of dietary RSM and FRSM on serum biochemical parameters of broiler chicks on day 42.

	Treatments						
Items^1^	CON	RSM	FRSM_33_	FRSM_66_	FRSM_100_	SEM^2^	*P*-value
Cholesterol, mg/dl	138.43^a^	126.81^a^	113.53^b^	110.20^b^	106.68^b^	4.35	0.0006
Triglycerides, mg/dl	95.18^a^	91.86^a^	65.29^b^	60.01^b^	58.26^b^	2.82	<0.0001
HDL-C, mg/dl	78.77^a^	70.64^a^	57.68^b^	61.03^b^	59.63^a^	3.12	0.38
VLDL-C, mg/dl	19.03^a^	18.37^a^	13.05^b^	12.02^b^	11.65^b^	0.56	<0.0001
LDL-C, mg/dl	43.19^a^	41.23^a^	30.35^b^	27.91^b^	22.99^b^	3.52	0.004
Total protein, g/dl	3.72	3.27	3.29	3.34	3.36	0.18	0.41
Glucose, mg/dl	263.37	253.32	252.59	253.07	255.14	3.25	0.16

Treatment groups included: CON: Control (basal corn-SBM diet); RSM: Rapeseed meal was replaced entirely with SBM in the control diet; FRSM_33_: Fermented rapeseed meal was replaced with rapeseed meal at 33.33 % in the RSM diet; FRSM_66_: Fermented rapeseed meal was replaced with rapeseed meal at 66.66 % in the RSM diet. FRSM_100_: Fermented rapeseed meal was replaced entirely with rapeseed meal in the RSM diet.

1 HDL-C, high density lipoprotein cholesterol; VLDL-C, very low-density lipoprotein cholesterol; LDL-C, low density lipoprotein cholesterol.

2 SEM, standard error of the means.

### Small intestinal morphology

Table 7 presents data on the effect of experimental treatments on small intestinal morphology. Broiler chickens fed diets in which SBM was replaced with FRSM exhibited significantly greater villus height and villus height to crypt depth ratio in both the duodenum and jejunum compared to other dietary treatments (*P* < 0.05). In the jejunum, all FRSM-fed groups also showed a significant reduction in crypt depth (*P* < 0.05). However, no significant differences were observed among treatments in villus height, crypt depth, or villus height-to-crypt depth ratio in the ileum (*P* > 0.05).

**Table 7:** Effects of dietary RSM and FRSM on small intestinal morphology of broiler chicks on day 42.

	Treatments						
Items^1^	CON	RSM	FRSM_33_	FRSM_66_	FRSM_100_	SEM^2^	*P*-value
Duodenum							
Villus height, μm	1110. 6^c^	1050.95^d^	1144.13^b^	1170.38^ab^	1190.05^a^	9.65	<0.0001
Crypt depth, μm	192.88	200.68	179.13	187.14	175.89	7.67	0.19
V/C	5.76^bc^	5.24^c^	6.48^ab^	6.27^ab^	6.83^a^	0.30	0.01
Jejunum							
Villus height, μm	912.63^bc^	901.10^c^	940.35^ab^	947.15^ab^	954.70^a^	11.25	0.01
Crypt depth, μm	165.55^ab^	170.02^a^	162.07^b^	140.77^c^	137.15^c^	1.55	<0.0001
V/C	5.51^c^	5.30^c^	5.80^b^	6.72^a^	6.96^a^	0.08	<0.0001
Ileum							
Villus height, μm	515.29	515.40	517.50	519.62	521.50	3.06	0.55
Crypt depth, μm	125.65	125.92	124.32	124.20	123.85	0.76	0.25
V/C	4.10	4.09	4.16	4.18	4.21	0.04	0.27

Treatment groups included: CON: Control (basal corn-SBM diet); RSM: Rapeseed meal was replaced entirely with SBM in the control diet; FRSM_33_: Fermented rapeseed meal was replaced with rapeseed meal at 33.33 % in the RSM diet; FRSM_66_: Fermented rapeseed meal was replaced with rapeseed meal at 66.66 % in the RSM diet. FRSM_100_: Fermented rapeseed meal was replaced entirely with rapeseed meal in the RSM diet.

1 V/C, Villus height/Crypt depth.

2 SEM, standard error of the means.

### Cecal short-chain fatty acid

The dietary treatments influenced the cecal concentrations of short-chain fatty acids in broiler chickens on day 42 (Table 8). Broilers fed diets in which SBM was completely replaced by FRSM exhibited the highest concentrations of butyric and lactic acids, both greater than those in the control and RSM groups (*P* < 0.05). Partial replacement of RSM with FRSM at the 66.66 % level also resulted in elevated butyric and lactic acid concentrations compared to the RSM and control diets (*P* < 0.05). Although the concentrations of other short-chain fatty acids, including acetic, propionic, and valeric acids, tended to increase with higher levels of FRSM inclusion, these differences were not statistically significant (*P* > 0.05).

**Table 8:** Effects of dietary RSM and FRSM on cecal short-chain fatty acids content of broiler chicks on day 42.

	Treatments						
Items	CON	RSM	FRSM_33_	FRSM_66_	FRSM_100_	SEM^1^	*P*-value
Acetic acid, *μ*mol/g	36.22	35.56	36.69	39.26	41.06	1.26	0.09
Butyric acid, *μ*mol/g	17.34^c^	16.92^c^	19.65^bc^	22.14^ab^	23.18^a^	0.75	<0.0001
Lactic acid, *μ*mol/g	1.70^c^	1.66^c^	2.28^b^	2.67^b^	3.19^a^	0.11	<0.0001
Propionic acid, *μ*mol/g	8.89	8.75	9.23	9.58	10.04	0.30	0.06
Valeric acid, *μ*mol/g	1.75	1.68	1.76	1.83	1.92	0.05	0.07

Treatment groups included: CON: Control (basal corn-SBM diet); RSM: Rapeseed meal was replaced entirely with SBM in the control diet; FRSM_33_: Fermented rapeseed meal was replaced with rapeseed meal at 33.33 % in the RSM diet; FRSM_66_: Fermented rapeseed meal was replaced with rapeseed meal at 66.66 % in the RSM diet. FRSM_100_: Fermented rapeseed meal was replaced entirely with rapeseed meal in the RSM diet.

1 SEM, standard error of the means.

### Apparent ileal digestibility of nutrients

Table 9 illustrates the effects of the experimental diets on apparent ileal nutrient digestibility in broiler chickens. Birds fed the diet in which SBM was completely replaced by FRSM showed the highest apparent ileal digestibility of crude protein and ether extract, with values significantly greater than those observed in the RSM-fed group (*P* < 0.05). These digestibility values were comparable to those recorded in the SBM control group. Moreover, a stepwise improvement in the digestibility of crude protein and ether extract was observed with increasing levels of FRSM inclusion (*P* < 0.05). In contrast, the lowest digestibility values for both nutrients were recorded in birds fed the RSM diet (*P* < 0.05). The digestibility of dry matter and organic matter, however, was not significantly influenced by the dietary treatments (*P* > 0.05).

**Table 9:** Effects of dietary RSM and FRSM on the apparent ileal digestibility of nutrients of broiler chicks on day 42.

	Treatments						
Items	CON	RSM	FRSM_33_	FRSM_66_	FRSM_100_	SEM^1^	*P*-value
Dry matter, %	66.23	62.84	64.37	65.12	66.75	1.17	0.19
Crude protein, %	76.44^a^	63.20^c^	68.24^bc^	70.05^b^	78.07^a^	1.40	<0.0001
Ether extract, %	81.02^a^	70.19^b^	72.95^b^	74.00^b^	79.36^a^	1.09	<0.0001
Organic matter, %	65.14	60.80	62.72	63.03	65.73	1.56	0.22

Treatment groups included: CON: Control (basal corn-SBM diet); RSM: Rapeseed meal was replaced entirely with SBM in the control diet; FRSM_33_: Fermented rapeseed meal was replaced with rapeseed meal at 33.33 % in the RSM diet; FRSM_66_: Fermented rapeseed meal was replaced with rapeseed meal at 66.66 % in the RSM diet. FRSM_100_: Fermented rapeseed meal was replaced entirely with rapeseed meal in the RSM diet.

1 SEM, standard error of the means.

## Discussion

In the present study, the complete substitution of SBM with RSM resulted in a significant reduction in WG and a deterioration in FCR throughout the entire growth period in broiler chickens. These findings are consistent with those reported by Wu et al. (2022), who similarly observed reduced FI and impaired body WG in broilers fed RSM-based diets relative to those receiving SBM-based control diets. As reviewed by Khajali & Slominski (2012), the negative impact of RSM on growth performance can be partly attributed to its higher crude fiber content relative to SBM. Elevated dietary fiber content resulting from substantial SBM replacement with RSM has been shown to suppress FI and compromise WG in broilers (Gopinger et al., 2014). Moreover, the inclusion of RSM in poultry diets is often limited due to its adverse effects on nutrient digestibility and metabolizable energy efficiency (Rabie et al., 2015). These limitations are primarily associated with the presence of anti-nutritional factors in RSM, such as phenolic compounds, phytic acid, and glucosinolates, which can reduce feed palatability and impair nutrient utilization, thereby negatively affecting growth performance (Chiang et al., 2010).

Microbial fermentation has been widely recognized as an effective strategy for reducing anti-nutritional factors in feed ingredients and improving growth performance in broiler chickens (Jazi et al., 2017; Sugiharto and Ranjitkar, 2019; Ashayerizadeh et al., 2024b). In the present study, the inclusion of FRSM, particularly when it fully replaced SBM, improved growth performance compared to the RSM diets. However, the growth performance of broilers fed FRSM remained inferior to those fed the SBM-based control diet. These results are in line with previous reports by Xu et al. (2011), who found that FRSM could partially replace SBM in duckling diets without compromising performance. Similarly, Wu et al. (2022) and Xu et al. (2012) reported better performance in broilers fed FRSM than in those receiving unfermented RSM. Our previous research showed that fermentation of RSM resulted in a 47 % reduction in crude fiber, a 68 % decrease in glucosinolates, an 83 % reduction in phytic acid, and a 58 % decline in tannins and phenolic compounds, alongside a 7 % increase in crude protein content (Ashayerizadeh et al., 2017), which may partly explain the observed performance improvements. Furthermore, the high lactic acid concentration in fermented feeds contributes to improved intestinal morphology (Soumeh et al., 2019) and supports gastrointestinal health and hygiene in poultry (Chen et al., 2013). Additionally, fermented feeds exhibit probiotic-like properties (Jazi et al., 2018a), and probiotic supplementation, whether via feed (Jazi et al., 2018b) or drinking water (Dastar et al., 2016), has been well-documented to enhance growth rate and feed efficiency in poultry.

In the evaluation of carcass characteristics, although Toghyani et al. (2009) reported no significant effect of RSM on carcass weight, the present study found that feeding broilers diets containing RSM reduced carcass yield and increased abdominal fat deposition. Supporting these findings, Payvastegan et al. (2013) demonstrated that inclusion of 20 g/kg RSM decreased breast muscle yield, likely due to anti-nutritional factors in RSM disrupting gut microbiota composition and impairing nutrient digestion and absorption. In contrast to RSM diets, replacing SBM with FRSM significantly improved carcass traits; broilers fed FRSM diets showed higher carcass yield and lower abdominal fat percentage, with the most favorable results observed in the group receiving full FRSM replacement. These improvements in carcass, breast, and thigh muscle yields may be attributed to the modulation of skeletal muscle protein metabolism by fermented feed, as suggested by Saleh et al. (2014). Furthermore, the observed increase in liver weight in broilers fed RSM diets compared to those receiving SBM may reflect hepatotoxic effects associated with glucosinolate hydrolysis products or endocrine disturbances, particularly involving thyroid function (McNeill et al., 2004; Toghyani et al., 2009). Although microbial fermentation is known to effectively reduce anti-nutritional factors (Alshelmani et al., 2016), liver hypertrophy was also observed in broilers fed FRSM. This effect may be linked to the capacity of fermented feeds to lower intestinal pH and increase lactic acid bacteria populations, thereby promoting bile salt deconjugation. The resulting disruption of enterohepatic circulation leads to increased fecal bile acid excretion, which stimulates hepatic conversion of cholesterol into bile acids. Consequently, serum and tissue cholesterol concentrations may decline, while compensatory hepatic enlargement (hepatomegaly) occurs, as previously reported by Kalavathy et al. (2010).

The gut microbiota plays a crucial role in host physiology by regulating nutrient metabolism, modulating immune function, and maintaining systemic health and homeostasis. In this study, replacing SBM and RSM with FRSM at all inclusion levels reduced pH in both the crop and ileum, decreased total anaerobic bacterial counts, increased LAB populations in the crop, and lowered ileal coliform counts, indicating an improved microbial balance in the gastrointestinal tract. Similarly, Chiang et al. (2010) reported that substituting SBM with FRSM in broiler diets increased the abundance of lactobacilli in the colon and cecal digesta compared to non-fermented diets. Gungor et al. (2021) also found that dietary supplementation with fermented grape seed enhanced *Lactobacillus* spp. populations in the ileum. Likewise, Alshelmani et al. (2016) observed that feeding broilers diets containing fermented palm kernel cake increased LAB populations and reduced *Enterobacteriaceae* counts in the intestine. Fermented feeds are typically characterized by elevated lactic acid concentrations and high LAB counts (Wu et al., 2022). The increased lactic acid content contributes to gastrointestinal acidification, reshaping the microbial ecosystem toward a more balanced and beneficial composition and promoting colonization by LAB (Ashayerizadeh et al., 2024b; Sugiharto and Ranjitkar, 2019). LAB further reinforce this acidified environment through the production of short-chain fatty acids, which act as natural antimicrobial agents and create an inhospitable environment for pathogenic bacteria such as *Salmonella* and coliforms (Jazi et al., 2019).

Serum cholesterol and triglyceride levels are key indicators of lipid metabolism and can be influenced by dietary and environmental factors. In the present study, the replacement of SBM and RSM with FRSM significantly reduced serum concentrations of cholesterol, triglycerides, LDL-C, and VLDL-C compared to diets containing RSM and SBM. Similarly, Payvastegan et al. (2013) reported that dietary inclusion of RSM alone had no significant effect on serum cholesterol, triglycerides, or HDL-C levels in broilers. Consistent with our findings, Hu et al. (2016) observed decreased serum triglyceride levels in broilers fed FRSM compared with those receiving SBM-based control diets. In a related study, Chen et al. (2013) demonstrated that fermented liquid feed reduced serum cholesterol levels in geese, attributing this effect to the inhibition of cholesterol synthesis by LAB. *Lactobacillus* species may reduce circulating cholesterol through multiple mechanisms, including assimilation of cholesterol into bacterial cell membranes, utilization of cholesterol for microbial metabolism, and interference with bile acid reabsorption in the small intestine, thereby enhancing fecal cholesterol excretion (Alaqil et al., 2022).

In the present study, dietary replacement of soybean meal SBM and RSM with FRSM significantly increased villus height and the villus height to crypt depth ratio in the duodenum and jejunum of broiler chickens. These morphological changes indicate an improved nutrient absorptive capacity and overall gut health (Jia et al., 2010), as increased villus height expands the surface area for nutrient absorption, and a higher villus height to crypt depth ratio reflects reduced epithelial cell turnover and enhanced intestinal integrity (Gopinger et al., 2014). The findings of this study are consistent with those of Wu et al. (2022), who reported similar improvements in intestinal morphology in broilers fed FRSM diets. Comparable enhancements in intestinal architecture have also been documented in broiler chickens fed other fermented plant protein sources, such as fermented sesame meal and fermented cottonseed meal (Hajimohammadi et al., 2020; Jazi et al., 2017). These observed changes may be attributed to microbial fermentation, which reduces anti-nutritional factors and enriches the feed with bioactive peptides and beneficial microorganisms (Sugiharto and Ranjitkar, 2019; Hu et al., 2016). The resulting improvements in intestinal morphology likely contributed to enhanced digestive efficiency and better growth performance in broilers fed FRSM-based diets.

Short-chain fatty acids, primarily produced through microbial fermentation of dietary carbohydrates and proteins in the cecum, play a vital role in poultry gastrointestinal physiology by supporting energy metabolism, promoting intestinal epithelial development, strengthening mucosal barrier integrity, and inhibiting pathogenic bacteria through luminal acidification (Shabani et al., 2019). In the present study, feeding broilers diets containing FRSM, in place of SBM or RSM, significantly increased cecal concentrations of butyric and lactic acids. The findings of the present study align with those of Fujiwara et al. (2009), who reported that dietary supplementation with 2 % *Bacillus subtilis*-fermented soybean significantly elevated total volatile fatty acids and acetic acid concentrations in broiler chicks. Similarly, Xie et al. (2017) observed increased levels of propionic and butyric acids in the cecum of piglets fed diets containing fermented SBM. Previous studies have shown that lactate produced by *Lactobacillus* spp. in the cecum promotes the growth of butyrate-producing bacteria, thereby increasing butyrate concentrations in the cecum (Meimandipour et al., 2010). The observed increase in short-chain fatty acid levels in the cecum is likely due to the low pH and acidic properties of fermented feeds, which create an environment favorable for the growth and activity of lactic acid bacteria, leading to enhanced lactic acid production (Sugiharto and Ranjitkar, 2019). In the present study, the increased concentrations of short-chain fatty acids in broilers fed FRSM were positively correlated with an increased population of beneficial bacteria.

The present study demonstrated that replacing SBM with FRSM significantly improved the apparent ileal digestibility of crude protein and ether extract in broilers compared to the RSM diet, with digestibility values comparable to those observed in birds fed the SBM-based control diet. These findings are consistent with previous studies reporting enhanced nutrient digestibility following FRSM supplementation (Chiang et al., 2010; Ahmed et al., 2014). Moreover, similar improvements have been observed with other fermented plant-based protein sources, such as cottonseed meal and palm kernel cake, where fermentation increased protein and amino acid digestibility (Ashayerizadeh et al., 2024a; Alshelmani et al., 2016). The observed improvements are likely attributed to the action of microbial enzymes (e.g., phytase, xylanase, cellulase) produced during fermentation, which facilitate the breakdown of complex macromolecules into smaller, more soluble and absorbable compounds (Olukomaiya et al., 2019). Additionally, fermentation reduces anti-nutritional factors (e.g., glucosinolates, phytic acid) and increases the release of peptides and free amino acids, thereby enhancing protein solubility and absorption efficiency (Sugiharto and Ranjitkar, 2019). Beyond chemical modification, the presence of LAB and other fermentative microbes may positively influence gut morphology, potentially increasing villus height and surface area for nutrient absorption.

## Conclusion

The findings of this study indicated that complete replacement of SBM with RSM adversely affected broiler growth performance, nutrient digestibility, intestinal morphology, and gut microflora composition. In contrast, substituting SBM with FRSM not only mitigated these negative effects but also conferred additional benefits, such as improved gut microbial balance, healthier intestinal morphology, and enhanced cecal short-chain fatty acid production, even relative to the SBM-based diet. Although growth performance in birds fed FRSM remained lower than in those fed SBM, the observed improvements in gut health and function suggest that FRSM may serve as a functional alternative to SBM by promoting gut integrity and supporting overall physiological stability.

## Disclosures

The authors declare that there is no conflict of interest.
